# Age And Other Individual Difference Variables Predict Ageism Awareness

**DOI:** 10.1093/geroni/igaf122.2196

**Published:** 2025-12-31

**Authors:** Carly Pullen, Julie Hicks-Patrick

**Affiliations:** West Virginia University, Morgantown, West Virginia, United States; West Virginia University, Morgantown, West Virginia, United States

## Abstract

One in every two people endorses ageist attitudes (WHO, 2021). Identifying the 50% who do and do not endorse such attitudes may reduce bias in Society. We examined beliefs about ageism among an age-diverse sample, including 231 US-based adults (51.9% female; Mage= 47.51, SD = 16; 67.5% WNH). A hierarchical regression examined how prominent an issue people believe ageism to be (M = 2.38 out of 4, SD = 0.88). In Step 1, age, sex, and race were entered into the model. Results were significant, F (3,227) = 9.13, p < .001, but only 10.8% of the variance in how prominent an issue people believe ageism to be was explained. Age and sex uniquely contributed to the model, (βage = .26, p< .001) and (βsex = .16, p < .05). In Step 2, hostile and benevolent ageism were added to the model to determine if a person’s attitude regarding older adults impacted their perception of ageism as a problem. Results were significant, F (5, 225) = 9.6.40, p < .001, with only 12.4% of the variance in how prominent an issue people believe ageism to be was explained. Lower hostile ageism uniquely contributed to the model (β = -0.12, p < .05), while benevolent ageism was nonsignificant (β = .07, p = .26). Results of the current study call attention to ageism as a problem and draw attention to the impact it has on older adults is a step forward in combatting stereotypes, prejudice, and discrimination.

